# Comparative evaluation of OpenAI O1 and human performance in higher order cognition

**DOI:** 10.1038/s41598-025-33629-9

**Published:** 2025-12-26

**Authors:** Ehsan Latif, Yifan Zhou, Shuchen Guo, Yizhu Gao, Lehong Shi, Matthew Nyaaba, Arne Bewerdorff, Xiantong Yang, Xiaoming Zhai

**Affiliations:** 1 AI4STEM Education Center, University of Georgia, Athens, GA USA; 2 National GENIUS Center, University of Georgia, Athens, GA USA; 3 School of Computing, University of Georgia, Athens, GA USA; 4 School of Teacher Education, Nanjing Normal University, Nanjing, Jiangsu China; 5 Department of Mathematics, Science, and Social Studies Education, University of Georgia, Athens, GA USA; 6 Department of Workforce Education and Instructional Technology, University of Georgia, Athens, GA USA; 7 School of Social Sciences and Technology, Technical University of Munich, Munich, Bavaria Germany; 8 Faculty of Psychology, Beijing Normal University, Beijing, China

**Keywords:** Mathematics and computing, Psychology, Psychology

## Abstract

This study evaluates the performance of OpenAI’s o1-preview model in higher-order cognitive domains, including critical thinking, systematic thinking, computational thinking, data literacy, creative thinking, logical reasoning, and scientific reasoning. Using established benchmarks, we compared the o1-preview models’ performance to human participants from diverse educational levels. o1-preview achieved a mean score of 24.33 on the Ennis-Weir Critical Thinking Essay Test (EWCTET), surpassing undergraduate (13.8) and postgraduate (18.39) participants (*z* = 1.60 and 0.90, respectively). In systematic thinking, it scored 46.1 ± 4.12 on the Lake Urmia Vignette, significantly outperforming the human mean (20.08 ± 8.13, *z* = 3.20). For data literacy, o1-preview scored 8.60 ± 0.70 on test “Use Data” dimension, compared to the human post-test mean of 4.17 ± 2.02 (*z* = 2.19). On creative thinking tasks, the model achieved originality scores of 2.98 ± 0.73, higher than the human mean of 1.74 (*z* = 0.71). In logical reasoning (LogiQA), it outperformed humans with 90% ± 10 accuracy versus 86% ± 6.5 (*z* = 0.62). For scientific reasoning, it achieved near-perfect performance (0.99 ± 0.12) on the TOSLS, exceeding the highest human scores of 0.85 ± 0.13 (*z* = 1.78). While o1-preview excelled in structured tasks, it showed limitations in problem-solving and adaptive reasoning. These results demonstrate the potential of AI to complement education in structured assessments but highlight the need for ethical oversight and refinement for broader applications.

## Introduction

Artificial Intelligence (AI) has made significant advances in recent years, particularly with the emergence of large language models (LLMs) like OpenAI’s o1-preview model^1^. These developments have created new opportunities for integrating AI into education, especially to enhance higher-order thinking skills that are essential for academic and professional success^2,3^. Higher-order thinking skills, such as critical thinking, systematic thinking, metacognition, logical reasoning, and collaborative problem-solving, are crucial for navigating the complexities of modern education and for developing cognitive abilities required in the 21st-century workforce^4,5^.

Research has underscored AI’s potential to improve problem-solving and cognitive skills, including critical thinking and data literacy, with significant implications for education and research^6,7^. Reviews and bibliometric analyses highlight a decade of progress in AI applications for education^3^. However, critical questions remain about the extent to which AI models can replicate or surpass human performance in higher-order thinking tasks, particularly at the graduate education level^2,8^.

Evaluations of the OpenAI o1-preview model show promise in addressing cognitive challenges, such as complex problem-solving and logical reasoning^9,10^. Despite this, concerns persist about the model’s ability to consistently perform tasks requiring deeper cognitive processes, such as metacognition^11^ and scientific reasoning^2^. Mixed results from studies on analogical reasoning in AI indicate some successes but also highlight challenges in achieving the nuanced thinking characteristic of human cognition^12,13^.

**Fig. 1:**
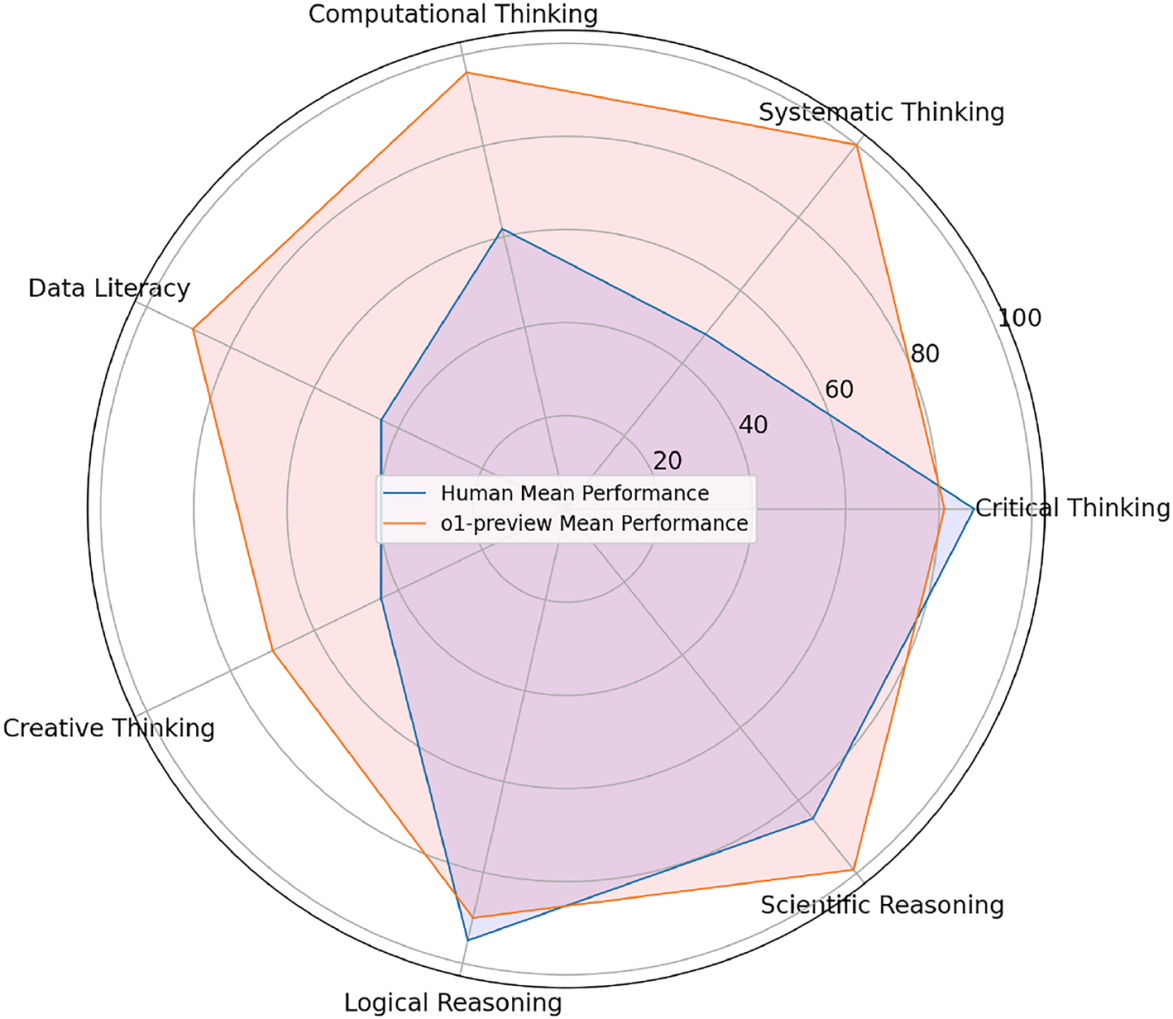
Performance overview of OpenAI o1-preview in higher-order thinking domains compared to human experts.

This paper addresses these gaps by comprehensively evaluating the o1-preview model across several key higher-order thinking domains, specifically *critical thinking*, *systematic thinking*, *computational thinking*, *data literacy*, *creative thinking*, *logical reasoning*, and *scientific reasoning*. These domains are identified as critical for graduate education and beyond^2–5^. Our findings reveal that the o1-preview model outperforms human experts in five out of seven domains, including systematic thinking, computational thinking, data literacy, creative thinking, and scientific reasoning (see Fig. 1 for an overview of its performance).

To provide conceptual grounding, we adopt a formal definition of higher-order thinking skills (HOTS) drawn from established educational and cognitive frameworks. Lewis and Smith^4^ describe HOTS as cognitive processes that occur “when individuals must interpret, analyze, or manipulate information,” extending beyond simple recall or routine application. Similarly, Anderson and Krathwohl’s revision^14^ of Bloom’s taxonomy positions HOTS within the upper levels of the cognitive hierarchy; *analyzing*, *evaluating*, and *creating*; processes that require flexible reasoning, synthesis of ideas, and metacognitive awareness. Reflective judgment models (e.g., King & Kitchener^15^) further emphasize the ability to evaluate evidence, manage uncertainty, and justify claims through reasoned argumentation.

Within this theoretical context, the seven instruments used in this study collectively operationalize the multidimensional nature of HOTS. The Ennis-Weir Critical Thinking^16^ Essay Test captures evaluative and argumentative reasoning; the complex systematic thinking task measures causal reasoning and multistep inference; the FLLIT computational thinking task assesses abstraction, algorithmic reasoning, and decomposition; the divergent thinking task represents creative ideation; the TOSLS evaluates data literacy and evidence-based judgment; the LogiQA instrument captures formal logical reasoning; and the LCTSR examines scientific reasoning and hypothesis testing. Together, these instruments span core components of HOTS as defined in contemporary cognitive and educational theory.

While the o1-preview model demonstrates effectiveness in computational tasks^2,3^, further research is needed to evaluate its capabilities in collaborative thinking and human-AI interactions in dynamic educational contexts^17,18^. Recent studies suggest that incorporating reflective and deconstructive strategies into AI systems can enhance their performance on complex, multi-step problems^11,19^. Additionally, the ethical implications of using AI in education, particularly in decision-making and reasoning tasks, demand careful consideration^20^.

In the following sections, we systematically evaluate the o1-preview model’s performance across these higher-order thinking domains, benchmarking its results against human participants. Our analysis provides insights into the potential and limitations of AI in graduate education, identifying areas where AI can contribute to the future of learning and teaching.

## Methods

### Participants

This research was conducted in accordance with approved guidelines, as this study did not involve the collection of new human subject data, and therefore institutional review board (IRB) approval was not required. All human benchmark data used in this research were obtained exclusively from previously published, peer-reviewed studies associated with each validated instrument. Importantly, only group-level summary statistics (e.g., reported means, standard deviations, normative distributions, or aggregated performance ranges) were used; no raw, individual-level, or identifiable human data were accessed or analyzed.

Comparisons between the o1-preview model’s performance and human benchmarks were computed using these published summary statistics. When normative means and standard deviations were available, AI performance was compared using z-scores or deviation scores relative to the reference population. In cases where summary-level test statistics were reported in the original studies, standard one-sample tests were applied to compare model outputs to the published group-level values. At no point did the analysis involve the reuse or reprocessing of human participants’ data. Accordingly, this study complies fully with ethical standards for secondary use of published data. The research involved no direct interaction with human participants, no access to raw datasets, and no use of sensitive or identifiable information.

### Instruments and datasets

This study employs well-established instruments and datasets tailored to each higher-order thinking category. For each domain, the performance of the o1-preview model is evaluated against the corresponding human cohort.

#### Critical thinking instruments

The Ennis-Weir Critical Thinking Essay Test (EWCTET)^21^ is a widely recognized tool designed to assess critical thinking skills through performance-based, context-driven written responses. Unlike standardized assessments, the EWCTET emphasizes real-world application, making it particularly effective for evaluating higher-order cognitive abilities. Studies such as those by Taghinezhad et al.^22^ highlight its capacity to assess cognitive complexity effectively. Developed by Robert Ennis and Eric Weir, the EWCTET challenges participants to construct, analyze, and evaluate arguments through coherent essays^16^.

The EWCTET uses scenario-based prompts, requiring participants to critically assess specific situations or arguments and present well-reasoned evaluations in written form^23^. It measures a range of critical thinking skills, including logical consistency, identification of assumptions, argument analysis, and evidence-based reasoning^24^. The tool’s accessibility and cost-effectiveness further contribute to its frequent use in educational research and practice.

The assessment typically takes 40 to 60 minutes, allowing participants sufficient time to construct thoughtful and nuanced arguments^21^. The scoring rubric evaluates multiple dimensions of critical thinking and written communication, including clarity, logical structure, and engagement with opposing viewpoints^24^.

Specific scoring criteria include:

- *Recognition of misuse of analogy, irrelevance, and circular reasoning:* Assesses the ability to identify logical fallacies.
- *Recognition of inadequate sampling and other logical fallacies:* Evaluates awareness of reasoning flaws, such as biased evidence.
- *Scoring for individual criteria:* Scores range from -1 to 3 points (poor to well-supported reasoning):
- *Ninth criterion:* Evaluates the overall quality of argumentation, including summarization and recognition of emotive language, with a maximum score of 5 points.

**Table 1:** Summary of human performance on the ennis-weir critical thinking essay test (EWCTET).

Authors	Participants	Assessment type	Mean score (SD)
Hatcher^25^	American freshmen at Baker University (four-year study)	Post-test after one-year compulsory critical thinking course	11.8–13.8 (SD not provided); Gains: 2.8–6.0
Davidson and Dunham^26^	36 Japanese EFL college students (treatment: n=17, control: n=19)	Post-test following critical thinking seminar for treatment group	Treatment: 6.6, Control: 0.6, $p <.001$
Hollis et al.^27^	100 online participants (63 females, 37 males), varied education levels	Post-test, no additional training	14.31 (SD = 8.45); Postgraduate: 18.39 (SD = 6.59), Undergraduate: 11.51 (SD = 8.23)

To evaluate the critical thinking performance of OpenAI’s o1-preview model, human performance data from studies using the EWCTET were established as a benchmark. These studies provide a comprehensive basis for assessing whether the model can match or exceed human critical thinking capabilities. Table 1 summarizes the participants, assessment types, and results from these studies.

#### Systematic thinking instruments

Numerous instruments have been developed to assess systematic thinking (ST), leveraging various theoretical frameworks across disciplines, using diverse methods, and targeting different educational levels. Common formats include mapping, interviews, scenario-based items, open-ended items, fill-in-the-blank items, and multiple-choice questions^28^.

In this study, we selected two ST instruments based on the following criteria:

1. To account for the limitations of the o1-preview model in identifying and generating images, we excluded instruments requiring respondents to create mappings or interpret indispensable visual information in prompts.
2. Research supports the use of scenario-based, ill-structured items as the most effective method for measuring ST. These items present realistic problems followed by a series of open- or close-ended questions to elicit ST skills^29^.
3. Since ST is extensively studied in biology and engineering^28,30^, we focused on theoretical frameworks and instruments developed in these fields, as they are more advanced and comprehensive.

*Human reference population* Human benchmark data for both the Village of Abeesee and Lake Urmia vignettes were drawn from published studies involving general-education undergraduate students. The original samples included approximately 120–150 participants enrolled in introductory social science or environmental studies courses at large public universities in the United States. Participants were non-experts with no specialized training in systems science, and the vignettes were administered under standard classroom or laboratory conditions following the procedures described in the source studies.

Based on these considerations, we selected two recently developed scenario-based, ill-structured instruments designed for higher education: the ”Village of Abeesee” instrument and the ”Lake Urmia Vignette” (LUV) instrument. These tasks were selected to evaluate participants’ ability to reason about multivariable systems, causal interactions, and potential ripple effects. For both instruments, all information necessary to analyze the scenarios was embedded directly within the vignette text. No external background knowledge was assumed or required. The original studies from which the human benchmark data were drawn designed these vignettes to be fully self-contained, ensuring that participants without specialized disciplinary training could engage with the tasks. To maintain equivalence across conditions, the o1-preview model was provided with the exact same vignette text used in the human studies, with no additional contextual prompts or supplementary knowledge. The model was explicitly instructed to rely only on the information provided within the scenario.

*Village of Abeesee Instrument* The Village of Abeesee instrument, developed by Grohs et al.^31^, is based on a framework that views ST as comprising three dimensions for general interdisciplinary use. This framework emphasizes the interconnectedness of technical and social aspects of modern problems and highlights the importance of stakeholder perspectives.

The instrument presents a scenario about the Village of Abeesee, which faces a complex issue regarding winter heating. Participants respond to six open-ended questions aligned with the framework’s dimensions. A pilot study was conducted to refine the scenario and gather qualitative data. Rubrics were developed through a multi-stage process using a pool of 93 student responses. The scoring dimensions include: Problem identification, Information needs, Stakeholder awareness, Goals, Unintended consequences, Implementation challenges, and Alignment. Each dimension is scored on a scale of 0 to 3, with a maximum possible score of 21.

*Lake Urmia Vignette (LUV) Instrument* The Lake Urmia Vignette (LUV) instrument, developed by Davis et al.^32^, is based on a theoretical framework that conceptualizes systems as webs of interconnected variables. Participants are presented with a scenario describing the real-world case of Lake Urmia’s desiccation. They are asked to describe the problem of Lake Urmia and explain why the lake shrank over the years.

The rubric evaluates participants’ responses by analyzing the number of variables, causal links, and feedback loops identified. During development, a pilot study and interviews were conducted with 30 graduate students to refine the instrument. This rigorous process ensures that the LUV instrument effectively captures participants’ ability to think systematically about socio-environmental issues.

To mitigate potential differences in prior knowledge across populations, comparisons were made only to human benchmark data derived from non-expert samples (e.g., general-education undergraduate students). As the tasks are constructed to evaluate reasoning about system structure rather than factual recall, performance differences are interpreted as reflecting systematic thinking processes rather than domain familiarity. These instruments are grounded in distinct theoretical frameworks and contextualized in engineering and socio-environmental systems.

#### Computational thinking instruments

Korkmaz et al.^33^ developed the Computational Thinking (CT) Skills instrument to assess college students’ computational thinking (CT) abilities across five dimensions: Creativity, Algorithmic thinking, Cooperation, Critical thinking, and Problem-solving. The CT instrument is a 29-item, five-point Likert scale ranging from 1 (strongly disagree) to 5 (strongly agree). It demonstrates high reliability, with a Cronbach’s alpha coefficient of 0.822 for the entire scale and 0.843, 0.869, 0.865, 0.784, and 0.727 for the five dimensions, respectively. This instrument has been widely adopted in research and has consistently yielded reliable human performance across various contexts.

*Human reference population* The human benchmark for the computational thinking task was based on data reported in the foundational validation study, which included 284 middle- and high-school students from the United States participating in computer science outreach and STEM enrichment programs. The sample represented novice to intermediate learners with varying levels of exposure to coding and algorithmic reasoning. The instrument was administered in supervised classroom or workshop environments according to standardized procedures.

The Algorithmic Thinking Test for Adults (ATTA), developed by Lafuente Martínez et al.^34^, is another validated tool designed to evaluate adults’ CT skills. It focuses on key CT components, including: Problem decomposition, Algorithmic thinking, Abstraction, Pattern recognition, and Debugging/evaluation. The ATTA consists of 20 items, including nine open-ended and 11 multiple-choice questions, offering a comprehensive assessment of computational thinking in adults.

#### Data literacy instruments

Data literacy has been explored across diverse fields, focusing on how individuals collect, analyze, and interpret data to make informed decisions in various settings^35,36^. It involves not only understanding complex information but also effectively addressing real-world challenges. Existing data literacy assessments can be categorized into two approaches: self-reflective and objective measures^37^.

Self-reflective approaches measure individuals’ self-reported data literacy competencies through surveys, questionnaires, semi-structured interviews, and think-aloud interviews^37^. Participants reflect on their data-related behaviors, practices, and attitudes. For example, a self-efficacy item might ask participants to rate their confidence in Use Data to identify students with special learning needs^38^.

Objective measures assess data literacy using test questions in three formats: conventional tests (multiple-choice and constructed-response questions), digital game-based assessments, and participation observations^37^.

To compare the data literacy of OpenAI o1-preview with humans, specific criteria were applied to select appropriate assessment instruments:

- *Assessment format* Standardized assessments were prioritized to ensure consistency in data literacy measurement.
- *Validation* Only empirically validated assessments with established reliability and validity were included.
- *Item type* To account for OpenAI o1-preview’s limitations with interactive and video-based content, selected assessments were restricted to conventional formats such as multiple-choice and constructed-response questions.
- *Audience* The assessments were targeted at adults, specifically post-secondary students, to align with the study’s focus population.

*Selected Instruments* Based on these criteria, two data literacy instruments were chosen for comparison:

*Merk et al.’s Data Literacy Test* Merk et al.^39^ developed a test to assess pre-service teachers’ data literacy, focusing on two major components: (1) *Use Data* (11 items): Evaluates understanding of data properties, manipulation, aggregation, and knowledge of statistics and psychometrics. (2) *Transform Data into Information* (11 items): Assesses the interpretation of data, use of data displays and visual representations, application of statistical methods, and summarization of data. Four items measure both components. The test was validated through exploratory and confirmatory factor analyses, reliability analysis (demonstrating high reliability with Cronbach’s $\alpha$), and concurrent criterion validity (correlation with state achievement test scores).

For the Merk et al.^39^ Data Literacy Test, only a subset of items could be administered to the *o1-preview* model because several of the original tasks relied on graphical displays, interactive data tables, or layout-dependent visual elements that could not be converted into text while preserving their cognitive demands. Accordingly, 9 of the 11 “Use Data” items and 5 of the 11 “Transform Data into Information” items were retained, excluding those that required direct visual inspection or manipulation. All retained items were reformulated into standardized text-based descriptions, and the model was evaluated on the full subset. To maintain comparability with published human benchmarks, scores were normalized to the proportion of items answered correctly within each subscale. Although the item sets were reduced, the retained items continue to represent the core conceptual dimensions of Merk et al.’s original framework, including data retrieval, interpretation, transformation, and inferential reasoning.

*Chen et al.’s Data Literacy Assessment* Chen et al.^40^ designed an assessment emphasizing the importance of data literacy for 21st-century citizens. It comprises three dimensions: *Data Management:* Covers data organization (1 item) and data manipulation (2 items). *Data Visualization:* Includes frequency distributions (2 items) and the use of visual charts (4 items). *Basic Data Analysis:* Focuses on central tendency (5 items), variability (3 items), and percentage calculations (1 item).

*Human reference population* The human comparison group for the Merk et al.^39^ and Chen et al.^40^ Data Literacy Test consisted of 569 and 435 respectively, undergraduate/graduate students from multiple U.S. institutions as reported in the original validation study. It is important to note that the reference to eye-tracking measures pertains only to the original validation study by Chen et al.^40^; no eye-tracking or physiological data were collected or analyzed in the present study. For the AI evaluation, all assessment items including those originally involving visual or graphical prompts were converted into standardized text-based descriptions. The model received each item in a stateless session with no prior context, using a uniform instructional prompt (e.g., “You will be presented with a scientific literacy question. Provide the best possible answer and explain your reasoning.”). All 28 items from the Chen et al.^40^instrument were administered independently, ensuring consistency with the benchmark task structure while maintaining methodological transparency.

Psychometric validation included item-total correlation and item response theory analyses. Eye-tracking studies further confirmed item validity by identifying differences in cognitive effort between successful and unsuccessful students. This study uses the data literacy assessments developed by Merk et al.^39^ and Chen et al.^40^ to evaluate the performance of OpenAI o1-preview. Human performance data reported in these studies serve as benchmarks for comparison, providing a basis for assessing the model’s data literacy competencies.

#### Creative thinking instruments

Creative thinking is commonly defined in two dimensions: divergent thinking and convergent thinking^41^.

*Divergent thinking* Divergent thinking involves solving problems or making decisions by employing strategies that deviate from commonly used or previously taught methods^42^. One of the most widely used tests for divergent thinking is the Alternate Uses Task (AUT)^43^, where participants generate original uses for common objects. Responses to the AUT are traditionally evaluated along four dimensions: *Fluency:* The number of ideas generated. *Flexibility:* The diversity of idea categories. **Originality:** The uniqueness of the ideas. *Elaboration:* The level of detail in the ideas.

Among these dimensions, originality is considered the most critical indicator of divergent thinking^44,45^. For example, the originality of AUT responses can be evaluated using automated AI scoring tools, such as the one developed by Organisciak et al.^44^, which has demonstrated high reliability and validity. This tool addresses the inefficiencies and subjectivity associated with traditional consensual assessment methods^44^.

The AUT, originally developed by Guilford^43^, has been extensively used among undergraduate, graduate, and post-graduate student groups, demonstrating good reliability and validity^46–48^. These qualities make it a suitable measure of divergent thinking for this study. The AUT used here includes three items: participants are asked to generate unconventional uses for a paperclip, a brick, and a can.

*Human reference population* Benchmark values for the divergent thinking task were drawn from samples of undergraduate students (typically $n \approx 100$–200) recruited from general-education psychology courses at North American universities. Participants completed standard Alternative Uses Task (AUT) prompts under timed laboratory or classroom conditions. Published norms report typical fluency, flexibility, and originality distributions for this population, which serve as the basis for comparison.

*Convergent thinking* Convergent thinking refers to the ability to use given clues to arrive at a single correct solution. A classic test for this dimension is the Remote Association Test (RAT), which asks participants to find a common link between three seemingly unrelated words^49^. For instance, the goal for the words ”SAME,” ”TENNIS,” and ”HEAD” is to identify a linking word, such as ”MATCH,” which forms compound words or semantic relationships (e.g., ”MATCH HEAD,” ”TENNIS MATCH”). The number of correct answers reflects the participant’s convergent thinking ability.

Originally developed by Mednick^49^, the RAT has since been translated into various languages (e.g., Chinese, Spanish) and validated across different populations, demonstrating good reliability and validity among college students^50,51^. For this study, the Chinese version of the RAT was selected to align with the language background of the participants. This version consists of 10 items, with a maximum score of 10. Higher scores indicate greater convergent thinking ability.

The AUT and RAT were chosen for their established validity and widespread recognition as classic tasks for measuring divergent and convergent thinking, respectively. These instruments provide complementary insights into the creative thinking process, making them well-suited for evaluating the creative thinking capabilities of OpenAI o1-preview and human participants.

#### Logical reasoning instruments

To evaluate the logical reasoning capabilities of the o1-preview model, we utilized the LogiQA dataset^52^. The LogiQA dataset comprises logical comprehension questions from the National Civil Servants Examination of China, designed to assess candidates’ logical thinking and problem-solving abilities. It contains 867 paragraph-question pairs categorized into five types of deductive reasoning, as defined by Hurley^53^:

*Categorical reasoning:* Determines whether a concept belongs to a specific category, often involving quantifiers such as ”all,” ”everyone,” ”any,” ”no,” and ”some”^54^.

*Sufficient conditional reasoning:* Based on conditional statements of the form ”If $P$, then $Q$,” where $P$ serves as the premise and $Q$ as the outcome^53^.

*Necessary conditional reasoning:* Involves statements such as ”P only if Q” or ”Q whenever P,” indicating that $Q$ is a necessary condition for $P$^53^.

*Disjunctive reasoning:* Uses premises in an ”either...or...” format, where the conclusion holds if at least one premise is true^53^.

*Conjunctive reasoning:* Features premises connected by ”both...and...” statements, where the conclusion is valid only if all premises are true^53^.

The dataset is divided into training (80%), development (10%), and testing (10%) sets. Among machine learning models, RoBERTa^55^ achieved the highest performance, with an accuracy of 35.31%, significantly below the human ceiling of 95.00%.

The LogiQA dataset serves as a robust benchmark for logical reasoning, enabling direct comparison of the o1-preview model’s performance with both human participants and existing machine learning models.

#### Scientific reasoning instruments

Numerous instruments are available for assessing scientific reasoning. Opitz et al.^56^ conducted a comprehensive review identifying 38 scientific reasoning tests, 14 of which were specifically designed for the university level. In this study, we focus on multiple-choice (MC) test instruments due to their advantages in standardized testing, which eliminates the need for an objective rater. Although MC formats are often critiqued for providing limited qualitative insights, they enable consistent assessment across a broad population.

The review by Opitz et al. identifies eight dimensions of scientific literacy: problem identification (PI), questioning (Q), hypothesis generation (HG), evidence generation (EG), evidence evaluation (EE), drawing conclusions (DC), communicating and scrutinizing (CS), and other skills (OT). Among the five MC instruments designed for university-level use, the Test of Scientific Literacy Skills (TOSLS) stands out for its ability to assess five of these dimensions (EG, EE, DC, CS, and OT). In contrast, other instruments cover only three dimensions each. Additionally, TOSLS is domain-specific, with a primary focus on biology, making it particularly relevant for evaluating scientific reasoning in specific contexts.

Test of Scientific Literacy Skills (TOSLS)

The 28-item TOSLS assesses nine skills related to scientific literacy:

1. Identify valid scientific arguments.
2. Evaluate the validity of sources.
3. Evaluate the use and misuse of scientific information.
4. Understand elements of research design and their impact on findings and conclusions.
5. Create graphical representations of data.
6. Read and interpret graphical representations of data.
7. Solve problems using quantitative skills, including probability and statistics.
8. Understand and interpret basic statistics.
9. Justify inferences, predictions, and conclusions based on quantitative data.

Gormally et al.^57^ designed TOSLS with a focused interpretation of scientific literacy that aligns closely with the concept of scientific reasoning^56,58^. The test’s internal reliability, measured using the Kuder-Richardson Formula 20 (KR-20)^59^, was reported as 0.73, meeting the acceptable threshold of 0.70^60^. Principal component analysis revealed a single-factor structure, supporting the test’s internal consistency.

*Human reference population* The human reference group for the Test of Scientific Literacy Skills (TOSLS) came from the widely used dataset in the original and subsequent large-scale validation studies. Typical samples range from 300 to over 1,000 high-school and undergraduate students across the United States, representing varied academic backgrounds. Testing was conducted in classroom settings as part of instruction in introductory science or pre-service teacher education courses.

Since its development, TOSLS has been widely used to assess scientific reasoning skills in university students across various studies^61–63^. The test’s focus on standardized measurement, broad coverage of scientific literacy, and domain-specific relevance makes it well-suited for evaluating OpenAI’s o1-preview model’s capacity for scientific reasoning.

### Procedure

For each domain, tasks were presented to the o1-preview model as text-input prompts. The model’s responses were evaluated using the same criteria applied to human participants, following the scoring guidelines of the respective instruments. Human participant data were drawn from existing studies to ensure consistency. Tasks were carefully matched in content and difficulty across human and AI cohorts to maintain comparability. Statistical analyses were conducted to evaluate significant differences in performance between the o1-preview model and human participants, with a particular focus on graduate-level performance as a benchmark.

*Independence and standardization of trials* For each cognitive domain, ten fully independent trials were executed. Each trial was conducted as a stateless API call to the o1-preview model, with no conversational context or memory carried over from any previous run. No trial occurred within an ongoing session; each was initialized in isolation to eliminate contextual carryover effects. All trials were generated using the same procedure, the same system prompt, and the same model snapshot available at the time of data collection.

The model was run using a fixed temperature parameter of $T = 0.0$, ensuring deterministic behavior and reducing stochastic variability in reasoning chains. Random seeds are not user-configurable in the o1-preview API; however, the use of temperature zero minimizes randomness and supports reproducibility. All other parameters were left at default OpenAI settings.

*Prompt structure and examples* All tasks were presented to the model using standardized text-based prompt templates. Each prompt included (i) a brief task label, (ii) the complete instrument question or scenario, and (iii) a direct instruction to “provide only the final answer” or “show reasoning then provide a final answer,” depending on the requirements of the assessment.

Representative prompt templates for each instrument (Critical Thinking, Systematic Thinking, Computational Thinking, Creative Thinking, Data Literacy, Logical Reasoning, and Scientific Reasoning) are provided in the Supplementary Information. These templates reproduce the structure of the original test items as closely as possible.

*Reformulation of visual items* Some items from the TOSLS and related assessments originally relied on visual representations (e.g., graphs, scatterplots, or data visualizations). To enable evaluation by a text-only model interface, these items were reformulated into descriptive text. For each graph, the axes, units, trend direction, and relevant numerical values were translated into a verbal description that preserved the interpretive demands of the original item (e.g., “The horizontal axis represents time in days; the vertical axis represents dissolved oxygen concentration.”). No inferential structure or required interpretation was altered. While full psychometric validation of these conversions is beyond the scope of this study, the reformulations were developed to maintain fidelity to the reasoning required in the original item formats.

### Data analysis

The study compared the performance of OpenAI o1-preview with that of human participants across seven higher-order thinking assessments. Percentage accuracy in answering the tasks was calculated, and mean performance scores were computed for both human participants and the o1-preview model, based on ten trials for each domain. To ensure comparability across different dimensions, scores were standardized within each assessment. To ensure a fair and systematic comparison between the o1-preview model and human participants, each task or domain was administered to the model in ten independent trials, with results averaged to account for response variability. The textual prompts presented to the o1-preview model were closely matched in content and structure to the materials used in published human studies, following the original assessment rubrics. Where human participants received specific instructional interventions or training (such as a critical thinking course), this was documented and used as a benchmark for comparison. For the o1-preview model, both zero-shot and role-based prompt strategies were employed to simulate novice and more advanced responses; however, unlike human participants, the model did not undergo any iterative training or extended learning on the task materials themselves. While the number of task iterations was standardized between humans and the model, direct matching of time-on-task or duration was not possible due to differences in human and model response dynamics. This limitation is acknowledged in interpreting the results, and we encourage future studies to explore additional normalization strategies for cross-modal comparisons.

Standard deviations were reported to evaluate how the o1-preview model’s performance deviated from the human mean. A one-sample *t*-test was used to determine the statistical significance of differences between the o1-preview model and human performance. Results were supplemented with confidence intervals and effect sizes to provide a comprehensive understanding of the observed differences. This approach ensured a rigorous comparison of AI and human capabilities across all assessed domains.

All analyses were performed using outputs generated under a single, stable configuration of the o1-preview model. Trials were conducted using the same account credentials, identical API parameters, and without modification to the system prompt or environment. Because OpenAI deployments may vary across accounts or time, this choice reduces variance attributable to deployment-level differences. We acknowledge, however, that potential account-level or system-level contextual biases are inherent to closed-model APIs and represent a limitation for external replication.

## Results

### Critical thinking

The critical thinking abilities of OpenAI’s o1-preview were evaluated using the Ennis-Weir Critical Thinking Essay Test (EWCTET), a widely recognized tool for assessing critical thinking across diverse educational contexts. To establish robust benchmarks for comparison, we referenced human performance data from three foundational studies. These studies provide insights into critical thinking outcomes across varying instructional methods and educational levels, serving as a basis for evaluating o1-preview’s performance.

*Human benchmarks* Hatcher^25^: Conducted over four years at Baker University, this study involved American freshmen who completed a compulsory, year-long critical thinking course. Post-test scores ranged between 11.8 and 13.8, marking gains of 2.8 to 6.0 points from pre-test scores of 5.8 to 9.4. These results illustrate the impact of structured, long-term instruction in improving critical thinking skills, providing a benchmark for sustained human cognitive development.

Davidson and Dunham^26^: This study was conducted at a Japanese private women’s junior college and included 36 first-year EFL (English as a Foreign Language) students. Participants were divided into a treatment group (n=17), who received critical thinking instruction, and a control group (n=19), who received only intensive English instruction. After one year, the treatment group achieved a mean score of 6.6 on the EWCTET, significantly higher than the control group’s mean score of 0.6 ($p <.001$). This highlights the effectiveness of integrating critical thinking instruction, even within a language-learning context.

Hollis et al.^27^: This study involved 100 online participants recruited via social media and online study platforms, with no specific critical thinking intervention provided. Post-test scores on the EWCTET averaged 14.31 (SD = 8.45). Educational background significantly influenced performance ($p <.001$), with postgraduates scoring an average of 18.39 (SD = 6.59), compared to undergraduates with a mean score of 11.51 (SD = 8.23). This study underscores the positive correlation between educational attainment and critical thinking skills.

*o1-Preview model performance* The o1-preview model was tested using iterative prompt strategies. A zero-shot prompt was employed first, followed by role-based prompts instructing the model to respond as a college student and then as a postgraduate student. These strategies mirrored the human participant studies, where responses were scored using the EWCTET scoring rubric. The results can be seen in Table 2.

**Table 2:** o1-Preview performance across three prompt iterations on EWCTET.

Prompt strategy	Total score	Maximum score
Zero-shot prompt	20	29
Role-based prompt as college student	26	29
Role-based prompt as graduate student	27	29

*Comparison with human benchmarks* We focused on the highest mean scores reported for human participants after learning interventions. Undergraduate students achieved a maximum mean score of 13.8, while postgraduate students scored an average of 18.39. In comparison, o1-preview achieved a mean score of 24.33 across the three prompt strategies. The corresponding z-scores indicate o1-preview outperformed human participants (see Table 3).

**Table 3:** Comparison of human performance and o1-preview model performance on the EWCTET.

Participant category	Mean total score	o1-Preview mean score	Z-score
Undergraduate students (highest after treatment)	13.8	24.33	1.60
Postgraduate students	18.39	24.33	0.90

These results demonstrate that OpenAI’s o1-preview exceeds human benchmarks in structured critical thinking tasks as measured by the EWCTET. However, the findings raise important questions about the comprehensiveness of such instruments in capturing the full spectrum of human critical thinking skills. While o1-preview excels in structured tasks, human oversight is essential when using the model in educational contexts. The findings highlight o1-preview’s potential as a supplementary tool for critical thinking instruction and assessment but underscore the need for careful integration to address its limitations and ensure alignment with broader educational goals.

### Systematic thinking

The systematic thinking (ST) abilities of o1-preview were evaluated using two instruments: the Village of Abeesee and the Lake Urmia Vignette (LUV). Davis et al.^64^ reported the average performance of 263 undergraduate students on the Village of Abeesee instrument and 155 undergraduates on the LUV instrument. The mean scores and standard deviations for each dimension are presented in Table 4. To compare o1-preview’s performance with human participants, the tests were administered to o1-preview in 10 trials, with the mean score, standard deviation, and z-score for each dimension calculated and included in Table 4.

The results indicate that o1-preview outperformed the average human scores in all seven dimensions of the Village of Abeesee instrument, suggesting that the model performed better on average than undergraduate students. For the LUV instrument, o1-preview also achieved significantly higher mean scores across all three dimensions compared to the human participants. This demonstrates that o1-preview generally excels in systematic thinking when compared to undergraduate students.

According to the z-scores, o1-preview performed exceptionally well in the ”Feedback Loops” dimension of the LUV instrument, achieving the highest z-score (6.53). This indicates that o1-preview is particularly adept at identifying feedback loops, which involve interconnected causal relationships within complex systems.

**Table 4:** Overall performance of human and o1-preview on systematic thinking.

ST instrument	Dimension	Human score (mean ± SD)	o1-Preview (mean ± SD)	Z-score
The Village of Abeesee	Problem identification	1.62 ± 0.64	2.50 ± 0.62	1.38
	Information needs	1.81 ± 0.52	2.90 ± 0.21	2.10
	Stakeholder awareness	1.23 ± 0.99	2.95 ± 0.16	1.74
	Goals	1.71 ± 0.62	2.90 ± 0.21	1.92
	Unintended consequences	1.38 ± 0.58	2.65 ± 0.24	2.19
	Implemented challenges	1.64 ± 0.57	2.70 ± 0.35	1.86
	Alignment	1.71 ± 1.00	2.35 ± 0.41	0.64
The Lake Urmia Vignette (LUV)	Variables	10.95 ± 4.00	19.70 ± 1.57	2.19
	Causal links	9.17 ± 3.97	23.30 ± 2.21	3.56
	Feedback loops	0.16 ± 0.45	3.10 ± 1.10	6.53
	Total score	20.08 ± 8.13	46.10 ± 4.12	3.20

The findings highlight o1-preview’s exceptional capabilities in systematic thinking, particularly in identifying feedback loops, a critical aspect of complex systems thinking. While these results are promising, they also underscore the need for further research to explore whether the performance gap reflects the model’s inherent strengths or limitations in the instruments used. Additionally, although o1-preview’s performance surpassed that of undergraduates, systematic thinking in real-world applications often requires collaborative and contextualized reasoning, which the current assessment methods may not fully capture.

The results suggest that OpenAI’s o1-preview has the potential to serve as a valuable tool for enhancing systematic thinking skills, particularly in educational settings. However, its application should be accompanied by human oversight to ensure that its outputs align with the nuanced requirements of real-world problem-solving.

### Computational thinking

*Human and o1-Preview Performance on Computational Thinking Skills* Table 5 compares the performance of human participants and o1-preview on the Computational Thinking (CT) Skills instrument across overall CT skills and specific dimensions: creativity, algorithmic thinking, cooperativity, critical thinking, and problem-solving. Human performance data were synthesized from three studies:

1. Liu et al.^65^, which involved 341 college students.
2. Şahin et al.^66^, which included 25 gifted science teachers.
3. Liao et al.^67^, which examined 44 sophomore undergraduate students.

The overall human performance mean was 3.92 (SD = 0.52), slightly higher than o1-preview’s mean score of 3.84 (SD = 1.56), yielding a z-score of -0.15. However, o1-preview outperformed human participants in four dimensions: creativity, algorithmic thinking, cooperativity, and critical thinking. Notably, o1-preview’s performance in critical thinking was exceptional (M = 4.8, SD = 0.42), achieving a z-score of 1.38. Conversely, o1-preview scored poorly on problem-solving (M = 1.0, SD = 0.0), significantly lower than the human mean (M = 3.68, SD = 0.63), with a z-score of -4.25.

**Table 5:** Comparison of o1-preview model and human performance on CT skills.

CT dimension	Human overall	o1-Preview	Z-Score
CT skills	3.92 ± 0.52	3.84 ± 1.56	− 0.15
Creativity	4.18 ± 0.63	4.56 ± 0.73	0.60
Algorithmic thinking	4.30 ± 0.62	4.50 ± 0.67	0.32
Cooperativity	3.94 ± 0.78	4.50 ± 0.53	0.72
Critical thinking	3.93 ± 0.63	4.80 ± 0.42	1.38
Problem-solving	3.68 ± 0.63	1.00 ± 0.00	− 4.25

*Human and o1-preview performance on algorithmic thinking test for adults* The Algorithmic Thinking Test for Adults (ATTA)^34^ was used to further evaluate o1-preview’s algorithmic thinking skills. The model achieved a perfect score (M = 20, SD = 0) across all 20 items (11 multiple-choice and 9 open-ended) in five rounds of testing. Human performance on the ATTA varied significantly: experts achieved a mean score of 14.63 (SD = 3.81), while novices scored an average of 9.11 (SD = 3.81). Performance differences were also observed across academic disciplines, with social sciences scoring the lowest (M = 9.11, SD = 4.54) and mathematics scoring the highest (M = 15.70, SD = 3.71). In all cases, o1-preview’s performance exceeded that of human participants, achieving high z-scores (see Table 6).

**Table 6:** o1-Preview performance on the algorithmic thinking test for adults (ATTA).

Participant category	Human overall	o1-Preview	Z-score
Experts	14.63 ± 3.81	20.00 ± 0.00	1.41
Novices	9.11 ± 3.81	20.00 ± 0.00	2.86
Social Sciences	9.11 ± 4.54	20.00 ± 0.00	2.40
Mathematics	15.70 ± 3.71	20.00 ± 0.00	1.16
Physics	15.26 ± 3.92	20.00 ± 0.00	1.21
Engineering	14.37 ± 3.17	20.00 ± 0.00	1.78
Computer Science	14.00 ± 3.80	20.00 ± 0.00	1.58

o1-preview demonstrated significantly higher capabilities in creativity, algorithmic thinking, cooperativity, and critical thinking dimensions of CT. However, its exceptionally low performance in problem-solving highlights a potential limitation in adapting its capabilities to real-world, ill-structured problems. On the ATTA, o1-preview’s perfect scores across all items suggest exceptional algorithmic reasoning abilities, surpassing all human groups tested. These results underline o1-preview’s potential as a tool for supporting computational thinking but also emphasize the need for human oversight, particularly for problem-solving tasks.

### Data literacy

The data literacy capabilities of o1-preview were evaluated using two validated instruments: Merk et al.’s^39^ Data Literacy Assessment and Chen et al.’s^40^ Data Literacy Assessment. These instruments assess distinct dimensions of data literacy, allowing for a comprehensive comparison between o1-preview and human participants.

*Merk et al.’s data literacy assessment* Merk et al.^39^ reported the performance of 89 pre-service secondary teachers from a large university in southern Germany. The assessment measured performance across two dimensions: ”Use Data” and ”Transform Data into Information.” Pre- and post-test scores were reported to evaluate the impact of an instructional intervention on participants’ data literacy skills. The mean scores and standard deviations for human participants, along with o1-preview’s mean scores, standard deviations, and *z*-scores, are presented in Table 7.

o1-preview outperformed human participants in both dimensions of the assessment, achieving substantially higher scores. For the ”Use Data” dimension, o1-preview attained a mean score of 8.60 (SD = 0.70), compared to human pre-test and post-test scores of 3.28 (SD = 1.84) and 4.17 (SD = 2.02), respectively, with a *z*-score of 2.89 for the pre-test and 2.19 for the post-test. Similarly, for the ”Transform Data into Information” dimension, o1-preview scored a mean of 4.80 (SD = 0.42), surpassing human pre-test and post-test scores of 2.96 (SD = 1.21) and 4.04 (SD = 1.34), with a *z*-score of 1.52 for the pre-test and 0.57 for the post-test.

**Table 7:** Performance of o1-preview and human participants on Merk et al.’s Data literacy assessment.

Data literacy	Human pre-test	Human post-test	o1-Preview	Pre-test	Post-test
Use data (9 items)	3.28 ± 1.84	4.17 ± 2.02	8.60 ± 0.70	2.89	2.19
Transform data into information (5 items)	2.96 ± 1.21	4.04 ± 1.34	4.80 ± 0.42	1.52	0.57

Item counts for the Merk et al. subscales reflect the reduced text-only subset used in this study. Items requiring graphical inspection or interactive components were excluded; scores represent proportion correct within each retained subscale

*Chen et al.’s data literacy assessment* Chen et al.^40^ reported the performance of 170 post-secondary students (average age = 22.81, SD = 4.25) from the Faculty of Education at a western Canadian university. This assessment evaluates three dimensions of data literacy: ”Data Management,” ”Data Visualization,” and ”Basic Data Analysis.” The mean scores, standard deviations, and *z*-scores for human participants and o1-preview are provided in Table 8.

o1-preview scored higher than human participants across all three dimensions. For ”Data Management,” o1-preview achieved a mean score of 2.00 (SD = 0.30), compared to the human mean of 0.17 (SD = 0.44), resulting in a *z*-score of 4.16. For ”Data Visualization,” o1-preview achieved a perfect score of 6.00 (SD = 0.00), exceeding the human mean of 3.56 (SD = 1.46) with a *z*-score of 1.67. Finally, for ”Basic Data Analysis,” o1-preview scored 9.00 (SD = 0.00), surpassing the human mean of 5.38 (SD = 2.22) with a *z*-score of 1.63.

**Table 8:** Performance of o1-preview and human participants on Chen et al.’s data literacy assessment.

Data literacy	Human (mean ± SD)	o1-Preview (mean ± SD)	o1-Preview Z-score
Data management (3 items)	0.17 ± 0.44	2.00 ± 0.30	4.16
Data visualization (6 items)	3.56 ± 1.46	6.00 ± 0.00	1.67
Basic data analysis (9 items)	5.38 ± 2.22	9.00 ± 0.00	1.63

Human sample size: $N = 555$ (from the original study). AI results reflect the mean across 10 stateless trials. Scores represent (raw / normalized / percentage) values as indicated

In both assessments, o1-preview consistently outperformed human participants across all dimensions, indicating competitive capabilities in understanding, interpreting, and analyzing data. These findings suggest that o1-preview could serve as a valuable tool to support or enhance data literacy education, particularly in developing critical data interpretation and analytical skills. However, while the results are promising, future research should explore o1-preview’s performance in more complex, real-world data scenarios to ensure its applicability beyond structured testing environments.

### Creative thinking

Human creative thinking has been assessed in previous studies using both divergent and convergent thinking tasks^68,69^. Divergent thinking was evaluated using the Alternate Uses Task (AUT), in which participants generated as many creative uses as possible for common objects (e.g., a paperclip, brick, or can). Convergent thinking was measured using the Remote Associates Test (RAT), which assesses participants’ ability to find a common link between unrelated words.

*Human performance* Urban et al.^68^ assessed the divergent thinking abilities of 68 university students (N = 52, males = 22; primarily from social sciences and humanities) using the AUT. Participants provided creative uses for common objects, and originality was scored on a 5-point scale by trained experts. The average originality score was 1.74.

For convergent thinking, Xia et al.^69^ employed the RAT to assess 54 Chinese bilingual university students. High-proficiency bilingual participants (N = 27) scored significantly higher on the RAT (M = 27.38) compared to low-proficiency participants (M = 23.80; $p = 0.003$), demonstrating the impact of bilingual proficiency on convergent thinking abilities. These studies provide benchmarks for evaluating the creative problem-solving skills of university students.

*o1-Preview performance* The AUT was used to evaluate OpenAI o1-preview’s divergent thinking performance. The model was prompted to generate as many original uses as possible for common objects (e.g., a paperclip, brick, or can). Originality was scored using an automated AI-based scoring tool developed by Organisciak et al.^44^. o1-preview achieved an overall mean originality score of 2.98, higher than the human benchmark of 1.74 (see Table 9).

For convergent thinking, o1-preview was evaluated using a Chinese version of the RAT adapted from Xia et al.^70^. The model was presented with 10 Chinese word association problems and achieved a total score of 7 out of 10, reflecting an accuracy rate of 70%. This performance surpasses the accuracy rate of 44.12% observed in human participants in similar tasks.

*Comparison between human and o1-preview performance* Table 9 summarizes the comparative results for divergent and convergent thinking tasks. For divergent thinking, o1-preview’s mean originality score (2.98) was notably higher than the human average score (1.74) on the AUT. This indicates that o1-preview generates creative ideas with a higher degree of originality compared to university students. In the convergent thinking task, o1-preview achieved a 70% accuracy rate on the RAT, outperforming the human benchmark of 44.12%, suggesting robust performance in identifying commonalities among unrelated concepts.

**Table 9:** Comparison of human and o1-preview performance on creative thinking tasks.

Task	Human overall	o1-Preview	Z-score
Divergent thinking (AUT)	1.74 ± 0.71	2.98 ± 0.73	0.71
Convergent thinking (RAT)	44.12 ± 6.21	70.00 ± 10.00	1.67

The results highlight OpenAI o1-preview’s competitive edge in divergent thinking tasks, as evidenced by its higher originality score on the AUT compared to human participants. The model also performed well in convergent thinking, achieving higher accuracy rate on the RAT. These findings suggest that o1-preview has significant potential as a tool for enhancing creative thinking, especially in generating novel ideas and identifying relationships among concepts. However, further research is needed to evaluate the model’s performance in less structured, real-world creative tasks to ensure its practical applicability beyond controlled experimental settings.

### Logical reasoning

Logical reasoning refers to the ability to draw valid conclusions based on given premises, encompassing both deductive and inductive reasoning. It is a critical skill for addressing complex problems and is widely applied across various fields, including STEM education. Effective logical reasoning involves multi-step analysis and inference, enabling coherent conclusions. In the context of STEM tasks, logical reasoning not only allows models to follow explicit instructions but also empowers them to interpret and apply implicit knowledge, demonstrating the capacity for step-by-step reasoning.

*Human and o1-preview performance on LogiQA* The logical reasoning capabilities of o1-preview were evaluated using the LogiQA dataset, a benchmark dataset designed to assess logical comprehension and reasoning. LogiQA includes a range of logical reasoning tasks derived from real-world examinations and challenges participants to analyze premises, identify relationships, and draw valid conclusions.

Table 10 summarizes the performance of human participants and o1-preview on the LogiQA dataset. Human participants, represented by a sample size of 651, achieved an average accuracy of 86.00% (SD = 6.5%). In contrast, o1-preview demonstrated superior performance with an average accuracy of 90.00% (SD = 10%) over 10 trials. These results indicate that o1-preview not only matches but slightly surpasses human accuracy in logical reasoning tasks, showcasing its robust analytical and inferential capabilities.

**Table 10:** Comparison of human and o1-preview performance on LogiQA (accuracy %).

Model	Sample size	Accuracy (%)
Human	651	86.00 ± 6.50
o1-Preview	10	90.00 ± 10.00

The results highlight o1-preview’s high logical reasoning capabilities, as it achieved higher accuracy than human participants on the LogiQA dataset. This performance underscores the model’s potential to process complex, multi-step reasoning tasks effectively. However, it is important to note the difference in sample sizes between human participants and o1-preview trials, which may influence the robustness of the comparison. Additionally, while o1-preview excels in structured logical reasoning tasks, further evaluation is needed to assess its performance in more dynamic and less constrained real-world scenarios.

The findings suggest that OpenAI o1-preview demonstrates exceptional logical reasoning abilities, comparable to and surpassing human performance in structured tasks like those in the LogiQA dataset. This highlights the potential for integrating AI models into domains requiring rigorous logical reasoning, though careful consideration should be given to the variability and context of real-world applications.

### Scientific reasoning

Scientific reasoning, as measured by the Test of Scientific Literacy Skills (TOSLS), serves as a critical benchmark for assessing higher-order thinking skills in various educational contexts. Multiple studies have established human performance benchmarks on the TOSLS, reporting a range of mean scores across diverse student and professional cohorts.

*Human performance benchmarks* Gormally et al.^57^ reported mean scores (SD) ranging from 0.42 (0.15) for students from midsized state colleges to 0.85 (0.13) for students at private research universities. Biology experts employed at universities achieved the highest score of 0.91 (0.09). Suwono et al.^63^, using an adapted version of the TOSLS, observed scores between 0.33 and 0.69 among Indonesian pre-service biology teachers. Segarra et al.^61^ reported scores ranging from 0.55 to 0.63 for US undergraduate students enrolled in general education biology courses. Firdaus et al.^71^ noted weak performance across most TOSLS items among Indonesian pre-service biology teachers but did not report a combined score. Propsom et al.^62^, in a study involving 800 US university students, reported mean TOSLS scores ranging from 0.60 (SD = 0.17) for first-year students to 0.66 (SD = 0.20) for seniors. Science and math majors scored higher, with mean scores of 0.64 (SD = 0.17) in their first year and 0.74 (SD = 0.17) in their senior year.

*o1-Preview performance* OpenAI’s GPT-o1-preview achieved a near-perfect score of 0.99 (SD = 0.12) across five trials on the TOSLS, surpassing all student cohorts and even biology experts employed at universities. Only one question (Item 2) was answered incorrectly in two out of five trials. This item required identifying the graph that best represents given data, originally presented as four visual graph plots. To accommodate GPT-o1-preview’s text-only input limitations, the question was modified into a text-based format using GPT-4o. This modality shift may have contributed to the errors observed.

*Comparison of human and AI performance* Table 11 summarizes the highest mean TOSLS scores reported across different human cohorts and compares them to o1-preview’s performance. While human participants demonstrated a wide range of abilities depending on context and expertise, o1-preview consistently outperformed all groups, achieving the highest z-score of 1.78.

**Table 11:** Comparison of human and AI performance on the TOSLS.

Sample	Sample size	TOSLS score	Z-score
US private research university nonmajors^57^	50	0.85 ± 0.13	0.80
Indonesian pre-service biology students^63^	33	0.69 ± 0.10	− 0.33
US undergraduate biology students^61^	83	0.63 ± 0.11	-0.75
US science and mathematics majors^62^	81	0.78 ± 0.15	0.30
GPT-o1-preview (AI trials)	5	0.99 ± 0.12	1.78

The results highlight GPT-o1-preview’s exceptional performance in scientific reasoning tasks as assessed by the TOSLS. Its near-perfect score suggests a competitive ability to interpret and analyze scientific information, surpassing both students and professional experts. However, the modification of visual-based questions to text-based formats introduces potential biases, and further research is needed to evaluate the model’s performance in scenarios involving complex visual data. Additionally, while o1-preview excels in structured assessments, its applicability to open-ended or real-world scientific reasoning tasks warrants further investigation.

The comparison of scientific reasoning skills using the TOSLS demonstrates GPT-o1-preview’s superiority over human participants, achieving the highest reported scores across all cohorts. These findings underscore the potential of AI systems like GPT-o1-preview as tools for advancing scientific literacy and reasoning in educational and professional settings. Nevertheless, careful consideration is required to address the model’s limitations and ensure its effective integration into real-world applications.

## Discussion

This study examined the performance of the o1-preview model across multiple domains commonly associated with higher-order thinking skills (HOTS), drawing on established frameworks such as Anderson and Krathwohl’s revision of Bloom’s taxonomy^14^ and Lewis and Smith’s definition of HOTS as complex, non-routine cognitive processes^4^. When evaluated against published human benchmarks, the model performed competitively in several structured reasoning domains; particularly critical thinking, logical reasoning, and aspects of scientific literacy. At the same time, important weaknesses emerged, most notably in computational thinking problem-solving, where the model scored below human means. These results highlight a nuanced pattern that does not support blanket claims of AI superiority, but rather a mixed profile of strengths and limitations.

A key interpretive consideration is that the seven instruments used in this study do not measure a single unified construct. Instead, they assess distinct cognitive competencies often grouped under the broader HOTS umbrella. For example, the LCTSR evaluates scientific reasoning, whereas the TOSLS assesses scientific literacy^57^, a related but conceptually distinct construct focused on data interpretation and scientific communication. Systematic thinking, computational thinking, and creative thinking similarly represent different facets of complex cognition. Distinguishing these domains prevents conceptual conflation and clarifies that the model’s performance reflects variation across heterogeneous cognitive skills rather than a monolithic HOTS construct.

Across domains, the o1-preview model showed particular strength on tasks requiring structured, rule-based inference. Its high accuracy on logical reasoning tasks in LogiQA^52^ and strong performance on analytical items within TOSLS^57^ suggest a proficiency in pattern-based reasoning and text-driven evidence evaluation. In creative thinking tasks, the model generated responses with high originality scores in the divergent-thinking format of the Alternate Uses Task (AUT)^43,44^, indicating an ability to produce semantically varied and novel ideas within structured constraints. These findings align with observations that large language models excel in analytical tasks where structure, goal states, and response expectations are well specified.

In contrast, the model’s weaknesses emerged most clearly in open-ended, ill-structured domains. Its lower performance on computational thinking problem-solving items^33,34^ illustrates challenges in constructing stable, multi-step reasoning processes or algorithmic strategies when solutions require flexible coordination of subgoals. Similarly, systematic thinking tasks revealed inconsistencies in the model’s ability to integrate contextual cues or causal relationships beyond surface-level patterns. These limitations, which parallel known difficulties in LLM reflective judgment and meta-reasoning, underscore that current architectures remain limited in domains demanding domain knowledge, abstraction, or high-level cognitive regulation.

From a theoretical perspective, these results can be situated within established HOTS frameworks. Analytical and rule-based tasks, aligned with mid-level HOTS categories such as analysis and evaluation, were areas where the model performed strongly. By contrast, tasks requiring reflective judgment, contextual sensitivity, or adaptive problem-solving correspond to higher layers of cognitive complexity and were more challenging for the model. This asymmetry suggests that AI’s cognitive profile aligns with structured analytical components of HOTS but diverges from human performance in domains requiring flexible, metacognitive, or ill-structured reasoning.

The implications for educational practice should therefore be interpreted cautiously. Although the model’s strong performance in structured tasks indicates potential value as a supportive analytical tool, real-world educational environments frequently involve open-ended inquiry, collaborative dialogue, and context-dependent problem-solving, tasks that extend beyond text-based reasoning and into the socioemotional and situational dimensions of learning. AI systems may offer value in generating practice items, scaffolding analytical thinking, or offering step-by-step explanations, but their role remains limited in instructional settings requiring interpretive judgment, negotiation of meaning, or creative synthesis. As such, AI should be viewed as complementing, rather than replacing, human instructional expertise.

Reproducibility and generalizability are further considerations that constrain interpretation. Large language models exhibit inherent non-determinism, and outputs can vary across accounts, model snapshots, or deployment environments, even when identical prompts are used. Because o1-preview is a closed, continuously updated model, its performance may change over time in ways that are inaccessible to researchers. Moreover, several instruments in this study contained visual or graphical components that were converted into descriptive text for model compatibility; a modality mismatch that limits comparability with human participants. The reliance on standardized, text-based benchmarks also reduces ecological validity relative to authentic tasks involving multimodal stimuli, social interaction, or evolving problem states. These constraints require that generalizations remain tightly bounded by the controlled conditions of the present study.

Several limitations of the study also map directly onto the observed performance patterns. The model’s weakest domain; computational problem-solving; reflects broader architectural challenges in maintaining stable reasoning chains. The necessity of text-only reformulation for visual items likely influenced performance on scientific literacy tasks. Methodological factors such as stateless trial design, prompt formatting, and potential contextual biases in API-based systems influence reproducibility and should be considered when interpreting comparative results. These linked limitations emphasize that conclusions should be evaluated in the context of specific task structures and methodological constraints.

Taken together, the findings suggest that contemporary language models can approximate certain components of HOTS in structured, text-based environments, but do not yet exhibit the generalized, flexible, or context-sensitive reasoning characteristic of human higher-order cognition. Their strengths lie primarily in analytical and text-interpretive tasks, whereas weaknesses emerge in reflective judgment, abstraction, and open-ended reasoning. The study contributes to emerging theoretical understanding by clarifying which components of human higher-order thinking align with current AI capabilities and which remain beyond present architectures. Future work should incorporate multimodal assessments, real-world educational tasks, and longitudinal human–AI comparisons to more fully characterize the role of AI within complex cognitive and learning environments.

## Conclusion

This study provides an initial comparison of the o1-preview model’s performance across multiple domains of higher-order thinking, showing that while the model performs competitively with human benchmarks in structured, text-based tasks involving analytical and rule-based reasoning, it demonstrates clear limitations in open-ended problem-solving, computational reasoning, and contexts requiring flexible integration of prior knowledge. Interpreted within established HOTS frameworks, these results indicate that current language models approximate specific analytical components of higher-order cognition but do not exhibit broad, domain-general higher-order thinking. Given methodological constraints; including reliance on published summary statistics, text-only task formats, limited trial numbers, and inherent non-determinism in large language models; the findings should be viewed as preliminary and context-dependent. Overall, the study highlights both the potential and the boundaries of contemporary AI systems in relation to complex cognitive tasks, underscoring the need for future multimodal, replicable, and ecologically valid human–AI comparative research.

## Data Availability

The authors confirm that the data supporting the findings of this study are available within the article.

## References

[1] OpenAI. Introducing OpenAI o1-preview (2024).

[2] Zhai, X., Nyaaba, M. & Ma, W. Can generative AI and chatgpt outperform humans on cognitive-demanding problem-solving tasks in science? *Sci. Educ.* 1–22 (2024).

[3] Guo, S., Zheng, Y. & Zhai, X. Artificial intelligence in education research during 2013–2023: A review based on bibliometric analysis. *Educ. Inf. Technol.* 1–23 (2024).

[4] Lewis, A. & Smith, D. Defining higher order thinking. *Theory Into Pract.* **32**, 131–137 (1993).

[5] Collins, R. Skills for the 21st century: teaching higher-order thinking. *Curric. Leadersh. J.* **12**, 1–8 (2014).

[6] Zhai, X. Chatgpt user experience: Implications for education. *SSRN Electron. J.* Available at SSRN 4312418 (2022).

[7] Zhong, T. et al. Evaluation of OpenAI o1: Opportunities and challenges of AGI arXiv:2409.18486 (2024).

[8] Marino, R. Fast analysis of the OpenAI o1-preview model in solving random K-SAT problem: Does the LLM solve the problem itself or call an external sat solver? *arXiv preprint* arXiv:2409.11232 (2024).

[9] Hu, H., Shang, Y., Xu, G., He, C. & Zhang, Q. Can gpt-o1 kill all bugs? *arXiv preprint* arXiv:2409.10033 (2024).

[10] Lightman, H. et al. Let’s verify step by step. *arXiv preprint* arXiv:2305.20050 (2023).

[11] Renze, M. & Guven, E. Self-reflection in LLM agents: Effects on problem-solving performance. *arXiv preprint* arXiv:2405.06682 (2024).

[12] Qin, C. et al. Relevant or random: Can LLMs truly perform analogical reasoning? arXiv:2404.12728 (2024).

[13] Musker, S., Duchnowski, A., Millière, R. & Pavlick, E. Semantic structure-mapping in LLM and human analogical reasoning. *arXiv preprint* arXiv:2406.13803 (2024).

[14] Anderson, L. W. & Krathwohl, D. R. *A taxonomy for learning, teaching, and assessing: A revision of Bloom’s taxonomy of educational objectives: complete edition* (Addison Wesley Longman, Inc., 2001).

[15] King, P. M. & Kitchener, K. S. The reflective judgment model: Twenty years of research on epistemic cognition. *Pers. Epistemol.* 37–61 (2012).

[16] Werner, P. The Ennis-Weir critical thinking essay test: An instrument for testing and teaching. *J. Read.* **34**, 494–495 (1991).

[17] Zhang, J., Xu, X. & Deng, S. Exploring collaboration mechanisms for LLM agents: A social psychology view. *arXiv preprint* arXiv:2310.02124 (2023).

[18] Lykov, A. et al. Industry 6.0: New generation of industry driven by generative AI and swarm of heterogeneous robots. arXiv:2409.10106 (2024).

[19] Lingo, R., Arroyo, M. & Chhajer, R. Enhancing LLM problem solving with reap: Reflection, explicit problem deconstruction, and advanced prompting. *arXiv preprint* arXiv:2409.09415 (2024).

[20] Sorin, V., Glicksberg, B. S., Korfiatis, P., Nadkarni, G. N. & Klang, E. Ethical alignment of LLMs in healthcare: Does GPT-o1 adopt a deontological or utilitarian approach? *medRxiv* (2024).

[21] Ennis, R. *Cornell Critical Thinking Tests Level X & Level Z Manual* (Midwest Publications, 1985).

[22] Taghinezhad, A., Riasati, M. J., Rassaei, E. & Behjat, F. The impact of teaching critical thinking on Iranian students’ writing performance and their critical thinking dispositions. *Brain Broad Res. Artif. Intell. Neurosci.* **9**, 64–80 (2018).

[23] Prayogi, S., Yuanita, L. & Wasis. Critical-inquiry-based-learning: Model of learning to promote critical thinking ability of pre-service teachers. In *Mathematics, Informatics, Science and Education International Conference (MISEIC)*, vol. 947 of *Journal of Physics Conference Series*, 10.1088/1742-6596/947/1/012013 (2018).

[24] Seker, H. & Komur, S. The relationship between critical thinking skills and in-class questioning behaviours of English language teaching students. *Eur. J. Teach. Educ.* **31**, 389–402. 10.1080/02619760802420784 (2008).

[25] Hatcher, D. L. Stand-alone versus integrated critical thinking courses. *J. Gen. Educ.* **55**, 247–272. 10.2307/27798054 (2006).

[26] Davidson, B. W. & Dunham, R. L. Assessing EFL student progress in critical thinking with the Ennis-Weir critical thinking essay test. In *Annual International Conference of the Japan Association for Language Teaching, 21st, Nagoya, Japan and International Conference on Critical Thinking and Educational Reform, Rohnert Park, CA* (Hokusei Gakuen University and Tezukayama College, 1996).

[27] Hollis, H., Rachitskiy, M., van der Leer, L. & Elder, L. Validity and reliability testing of the international critical thinking essay test form a (ICTET-A). *Psychol. Rep.* (in press). Forthcoming.

[28] Dugan, K. E., Mosyjowski, E. A., Daly, S. R. & Lattuca, L. R. Systems thinking assessments: Approaches that examine engagement in systems thinking. In *2021 ASEE Virtual Annual Conference Content Access* (2021).

[29] Norris, M. B., Grohs, J. R. & Knight, D. B. Investigating student approaches to scenario-based assessments of systems thinking. In *Frontiers in Education*, Vol. 7, 1055403 (Frontiers Media SA, 2022).

[30] Li, R. & Li, G. Developing and validating a biological system thinking test for middle school students. *Int. J. Sci. Math. Educ.* 1–21 (2024).

[31] Grohs, J. R., Kirk, G. R., Soledad, M. M. & Knight, D. B. Assessing systems thinking: A tool to measure complex reasoning through ill-structured problems. *Think. Ski. Creat.* **28**, 110–130 (2018).

[32] Davis, K. et al. The Lake Urmia vignette: a tool to assess understanding of complexity in socio-environmental systems. *Syst. Dyn. Rev.* **36**, 191–222 (2020).

[33] Korkmaz, Ö., Çakir, R. & Özden, M. Y. A validity and reliability study of the computational thinking scales (CTS). *Comput. Hum. Behav.* **72**, 558–569 (2017).

[34] Lafuente Martínez, M., Lévêque, O., Benítez, I., Hardebolle, C. & Zufferey, J. D. Assessing computational thinking: Development and validation of the algorithmic thinking test for adults. *J. Educ. Comput. Res.* **60**, 1436–1463 (2022).

[35] Koltay, T. Data literacy for researchers and data librarians. *J. Librariansh. Inf. Sci.* **49**, 3–14 (2017).

[36] Wolff, A., Gooch, D., Montaner, J. J. C., Rashid, U. & Kortuem, G. Creating an understanding of data literacy for a data-driven society. *J. Community Inform.* **12** (2016).

[37] Cui, Y., Chen, F., Lutsyk, A., Leighton, J. P. & Cutumisu, M. Data literacy assessments: A systematic literature review. *Assess. Educ. Princ. Policy Pract.* **30**, 76–96 (2023).

[38] Reeves, T. D. & Chiang, J.-L. Effects of an asynchronous online data literacy intervention on pre-service and in-service educators’ beliefs, self-efficacy, and practices. *Comput. Educ.* **136**, 13–33 (2019).

[39] Merk, S., Poindl, S., Wurster, S. & Bohl, T. Fostering aspects of pre-service teachers’ data literacy: Results of a randomized controlled trial. *Teach. Teach. Educ.* **91**, 103043 (2020).

[40] Chen, F. et al. Validating a novel digital performance-based assessment of data literacy: Psychometric and eye-tracking analyses. *Educ. Inf. Technol.* **29**, 9417–9444 (2024).

[41] De Rooij, A. & Vromans, R. D. The (dis) pleasures of creativity: Spontaneous eye blink rate during divergent and convergent thinking depends on individual differences in positive and negative affect. *J. Creat. Behav.* **54**, 436–452 (2020).

[42] Ashkinaze, J., Mendelsohn, J., Qiwei, L., Budak, C. & Gilbert, E. How AI ideas affect the creativity, diversity, and evolution of human ideas: Evidence from a large, dynamic experiment. *arXiv preprint* arXiv:2401.13481 (2024).

[43] Guilford, J. P. *The Nature of Human Intelligence* (McGraw-Hill, 1967).

[44] Organisciak, P., Acar, S., Dumas, D. & Berthiaume, K. Beyond semantic distance: Automated scoring of divergent thinking greatly improves with large language models. *Think. Ski. Creat.* **49**, 101356 (2023).

[45] Acar, S. et al. Applying automated originality scoring to the verbal form of torrance tests of creative thinking. *Gift. Child Q.* **67**, 3–17 (2023).

[46] Collins, M. J. D. A distracted muse: The positive effect of dual-task distraction on creative potential. *Creat. Res. J.* **32**, 357–367 (2020).

[47] Dumas, D. G. & Strickland, A. L. From book to bludgeon: A closer look at unsolicited malevolent responses on the alternate uses task. *Creat. Res. J.* **30**, 439–450 (2018).

[48] George, T. & Wiley, J. Fixation, flexibility, and forgetting during alternate uses tasks. *Psychol. Aesthet. Creat. Arts* **13**, 305 (2019).

[49] Mednick, S. The associative basis of the creative process. *Psychol. Rev.* **69**, 220 (1962). 14472013 10.1037/h0048850

[50] Li, L.-M., Luo, L.-L. & Liu, W. An objective measuring tool of creativity: The development of Chinese remote association test. *J. Northeast. Univ. (Soc. Sci.)* **17**, 19 (2015).

[51] Peláez-Alfonso, J. L., Pelegrina, S. & Lechuga, M. T. Normative data for 102 Spanish remote associate problems and age-related differences in performance. *Psicológica* **41**, 39–65 (2020).

[52] Liu, Z. et al. Logiqa: A challenge dataset for machine reading comprehension with logical reasoning. arXiv:2007.08124 (2020).

[53] Hurley, P. J. *A Concise Introduction to Logic* (Nelson Education, 2014).

[54] Abramsky, S. & Tzevelekos, N. Introduction to categories and categorical logic. In *New Structures for Physics*, Vol. 813 of *Lecture Notes in Physics* (ed Coecke, B.) , 3–94, 10.1007/978-3-642-12821-9_1 (Springer-Verlag, 2011).

[55] Liu, Y. et al. Roberta: A robustly optimized bert pretraining approach. *arXiv preprint* arXiv:1907.11692 (2019).

[56] Opitz, A., Heene, M. & Fischer, F. Measuring scientific reasoning-a review of test instruments. *Educ. Res. Eval.* **23**, 78–101 (2017).

[57] Gormally, C., Brickman, P. & Lutz, M. Developing a test of scientific literacy skills (TOSLS): Measuring undergraduates’ evaluation of scientific information and arguments. *CBE Life Sci. Educ.* **11**, 364–377 (2012). 23222832 10.1187/cbe.12-03-0026 PMC3516792

[58] Chen, Z. Milestones: cognitive. In *Encyclopedia of Infant and Early Childhood Development (Second Edition)* (ed Benson, J. B.) 330–338, 10.1016/B978-0-12-809324-5.21825-7 (Elsevier, 2020).

[59] Kuder, G. F. & Richardson, M. W. The theory of the estimation of test reliability. *Psychometrika* **2**, 151–160 (1937).

[60] Cronbach, L. J. Coefficient alpha and the internal structure of tests. *Psychometrika* **16**, 297–334 (1951).

[61] Segarra, V. A. et al. Student performance on the test of scientific literacy skills (TOSLS) does not change with assignment of a low-stakes grade. *BMC. Res. Notes* **11**, 1–5 (2018). 29970190 10.1186/s13104-018-3545-9 PMC6029066

[62] Propsom, P. M., Tobin, W. M. & Roberts, J. R. *Test of scientific literacy skills (TOSLS) indicates limited scientific thinking gains as a result of science and mathematics general education*. textitInterdiscip. Fac. Scholarsh. (2023).

[63] Suwono, H., Pratiwi, H., Susanto, H. & Susilo, H. Enhancement of students’ biological literacy and critical thinking of biology through socio-biological case-based learning. *J. Pendidik. IPA Indones.* **6**, 213–220 (2017).

[64] Davis, K. A. et al. Comparing self-report assessments and scenario-based assessments of systems thinking competence. *J. Sci. Educ. Technol.* **32**, 793–813 (2023).

[65] Liu, S., Peng, C. & Srivastava, G. What influences computational thinking? a theoretical and empirical study based on the influence of learning engagement on computational thinking in higher education. *Comput. Appl. Eng. Educ.* **31**, 1690–1704 (2023).

[66] Şahin, E., Sarı, U. & Şen, Ö. F. Stem professional development program for gifted education teachers: Stem lesson plan design competence, self-efficacy, computational thinking and entrepreneurial skills. *Think. Ski. Creat.* **51**, 101439 (2024).

[67] Liao, J. et al. *Scaffolding computational thinking with chatgpt*. *IEEE Trans. Learn. Technol.* (2024).

[68] Urban, M. et al. Chatgpt improves creative problem-solving performance in university students: An experimental study. *Comput. Educ.* **215**, 105031 (2024).

[69] Xia, T., An, Y. & Guo, J. Bilingualism and creativity: Benefits from cognitive inhibition and cognitive flexibility. *Front. Psychol.* **13**, 1016777 (2022). 36405189 10.3389/fpsyg.2022.1016777 PMC9670109

[70] Xia, T., Song, L., Wang, T. T., Tan, L. & Mo, L. Exploring the effect of red and blue on cognitive task performances. *Front. Psychol.* **7**, 784 (2016). 27303343 10.3389/fpsyg.2016.00784 PMC4880552

[71] Firdaus, L. et al. A quantitative study on the scientific literacy skills of prospective biology teachers. *J. Penelit. Pendidik. IPA* **9**, 80–86 (2023).

